# Novel Valorization of Fully Ripe Mangoes: A Dual Approach to Produce Prebiotic Short‐Chain Fructooligosaccharide–Enriched Products Through Enzymatic Synthesis and Osmotic Dehydration

**DOI:** 10.1155/ijfo/5590635

**Published:** 2026-05-21

**Authors:** Jariyaporn Changin, Warathep Buasum, Sirilux Chaijamrus, Monthana Weerawatanakorn, Tipawan Thongsook

**Affiliations:** ^1^ Department of Agro-Industry, Faculty of Agriculture, Natural Resources and Environment, Naresuan University, Phitsanulok, Thailand, nu.ac.th; ^2^ Department of Biology, Faculty of Science, Naresuan University, Phitsanulok, Thailand, nu.ac.th

## Abstract

Short‐chain fructooligosaccharides (sc‐FOS) are prebiotics with significant health benefits and potential as low‐calorie sweeteners. This study employed a dual strategy to valorize unmarketable, fully ripe “Nam Dok Mai” mangoes—commonly responsible for substantial postharvest losses—by converting their inherent sucrose into sc‐FOS and subsequently utilizing the sc‐FOS–enriched mango syrup for osmotic dehydration (OD) of mango pieces. sc‐FOS was successfully synthesized from mango juice using Pectinex Ultra SP‐L. The addition of exogenous sucrose further enhanced sc‐FOS production, resulting in a sucrose conversion of 67.54% (g FOS·100 g initial sucrose^−1^). Mango syrup derived from juice with added sucrose (JUICE SU) contained sc‐FOS of 58.07% (g FOS·100 g total sugar^−1^) and was used as the OD agent. OD at 60°C significantly increased the sc‐FOS uptake of mango pieces compared to dehydration at 40°C. The sc‐FOS–enriched mango syrup proved more effective in facilitating OD than conventional sucrose syrup, leading to greater solid gain and water loss. The OD mango pieces using sc‐FOS–enriched mango syrup as the OD agent contained sc‐FOS of approximately 30% (db). This research demonstrates a promising approach for transforming food waste into value‐added functional food products while mitigating economic losses and environmental impacts associated with unmarketable fully ripe mangoes.

## 1. Introduction

Short‐chain fructooligosaccharides (sc‐FOS) are a class of nondigestible carbohydrates composed of 2–4 *β*(2→1)‐linked fructosyl units with a terminal *α*‐D‐glucose. These compounds are of significant interest in the food industry due to their recognized prebiotic properties, which contribute to improved human health, particularly by stimulating the growth of beneficial bifidobacteria in the large intestine. Beyond their functional benefits, sc‐FOS also serve as low‐calorie, low‐glycemic sweeteners, offering a mild sweetness that integrates well with natural fruit flavors [1]. They are typically synthesized from sucrose via enzymatic transfructosylation using enzymes such as *β*‐fructofuranosidase (FFase) and fructosyltransferase (FTase). FFase and FTase are crucial as they catalyze the transfer of fructosyl units from sucrose to acceptor sugar molecules, forming sc‐FOS compounds such as 1‐kestose (GF2), nystose (GF3), and 1‐*β*‐fructofuranosylnystose (GF4) [2]. These enzymes are present in commercial preparations like Pectinex Ultra SP‐L and Viscozyme L, which are among the most commonly used enzymes in fruit juice industries for juice extraction. Commercial enzyme preparations with strong transfructosylation activity can efficiently produce sc‐FOS from sucrose [3]. The utilization of these commercial enzyme preparations offers a cost‐effective and efficient approach for large‐scale production, as they eliminate the need for complex enzyme purification processes while maintaining high catalytic stability and compatibility within existing food processing frameworks.

Although purified sucrose is an effective substrate for sc‐FOS synthesis, there is a growing interest in using natural raw materials to lower production costs and improve industrial sustainability. Recent studies have demonstrated the feasibility of using sucrose‐rich natural substrates for sc‐FOS synthesis. For instance, sugarcane syrup has been effectively used as a substrate for sc‐FOS production, taking advantage of its high sucrose content [2]. Cheng et al. [4] successfully converted sucrose in longan pulp into sc‐FOS using the commercial enzyme Viscozyme L, resulting in the production of prebiotic longan juice. Strawberry preparations have also been successfully employed for in situ enzymatic conversion of sucrose into sc‐FOS, leading to functional prebiotic products [5]. sc‐FOS produced from sucrose‐rich natural substrates offer multiple benefits beyond their prebiotic properties. For instance, when derived from sugarcane syrup, sc‐FOS not only enhance the functional value of the product but also contribute to higher antioxidant activity and a lower caloric content compared to pure sucrose [2].

Osmotic dehydration (OD) is an efficient food preservation technique that involves immersing fruit pieces in a hypertonic solution, thereby establishing a chemical potential gradient. This gradient facilitates water removal from the fruit while allowing solute uptake, enabling mass transfer without phase change. Compared to conventional drying methods, OD is considered more energy‐efficient and aligns with sustainable food processing technologies by reducing energy requirements. Furthermore, performing OD at low temperatures minimizes structural damage to plant tissues and helps preserve the nutritional and sensory attributes of the food [6]. OD is a common method for preserving tropical fruits like mango, conventionally using sucrose as the primary agent. Sucrose is traditionally favored due to its high solubility, low cost, and efficient water removal. However, increasing health concerns related to sucrose consumption, such as its cariogenic properties and high glycemic index, have led to a search for alternative sweeteners. Recent studies have explored promising alternatives. For instance, do Carmo et al. [7] investigated isomaltulose for osmotically dehydrating mango slices. Similarly, Jiménez‐Hernández et al. [8] explored inulin as another alternative sweetener for mango slices, also incorporating piquin‐pepper oleoresin to add functional benefits.

In recent years, juice concentrates—natural sources of nutritional and bioactive compounds—were utilized as alternative osmotic agents. The application of these concentrates facilitated a synergistic mass transfer process; specifically, the osmotic gradient led to the simultaneous dehydration of the food matrix and the infusion of health‐promoting solutes. This process resulted in the development of functional food products with enhanced nutritional and organoleptic characteristics. OD using concentrated juices allowed for the successful infusion of bioactive components, such as polyphenols and antioxidants, into the food matrix. Studies have shown that when using concentrated juices (e.g., chokeberry, flowering quince, and raspberry) for the OD of pumpkin, the bioactivity of the samples improved significantly, particularly in terms of polyphenol content and antioxidant activity [9]. Concentrated fruit juices were more effective than sucrose solutions in removing water during OD. For instance, concentrated grape juice was reported as an efficient osmotic agent for yellow melon, resulting in greater water loss (WL) and solid gain (SG) compared to sucrose [10]. The presence of glucose and fructose in most fruit concentrates provided a low‐molecular‐weight osmotic medium that penetrated cellular structures more readily, thereby enhancing WL and solute uptake [11, 12]. The incorporation of natural fruit concentrates has been shown to enhance the sensory characteristics of the final product. Substituting sucrose with bitter orange, apple, or grape concentrates in the OD of pomegranate seeds reduced sugar dependency while significantly enhancing sensory appeal through novel flavor profiles [13].

Postharvest losses of tropical fruits such as mangoes can be relatively high during the journey from farm to market. These losses are mainly caused by physical damage, rapid spoilage, and inadequate packaging and temperature management. These losses not only reduce marketable yield but also contribute to environmental and economic burdens. Mango (*Mangifera indica* L.), particularly the “Nam Dok Mai” cultivar, is naturally rich in sucrose and fructose, making it an excellent substrate for sc‐FOS production. Ripened mangoes also contain organic acids (e.g., malic and citric acids), volatile esters responsible for their characteristic aroma, and phytochemicals such as carotenoids [14]. These attributes position mango juice concentrate as a highly suitable and value‐added osmotic agent, offering both functional and sensory advantages in food processing.

This study proposed an innovative approach by integrating sc‐FOS production from ripe mangoes with their application in OD. To the best of our knowledge, this was the first investigation utilizing mango as a substrate for sc‐FOS synthesis and subsequently incorporating sc‐FOS into OD processes. This dual strategy not only introduced new opportunities for developing appealing fruit‐based products but also enabled modulation of the sugar profile in the final product, which may result in a higher prohealth potential. This study therefore focused on two key areas: first, investigating the production of sc‐FOS from fully ripe mango fruits, aiming to valorize these often‐underutilized fruits; and second, exploring the OD of mango pieces using sc‐FOS–enriched mango syrup. This exploration specifically evaluated how temperature affected the mango’s sugar composition, SG, and WL during dehydration.

## 2. Materials and Methods

### 2.1. Enzymes and sc‐FOS Standards

Pectinex Ultra SP‐L from *Aspergillus aculeatus* was purchased from Sigma‐Aldrich (St. Louis, Missouri, United States). sc‐FOS standards (1‐kestose, nystose, and 1F‐fructofuranosylnystose) were obtained from Megazyme (Lansing, Michigan, United States).

### 2.2. Raw Material Preparation

Fully ripe mangoes (*M. indica* L. cv. Nam Dok Mai) harvested in 2024 from Noen Maprang District, Phitsanulok Province, Thailand, were used in this study. Fruits were selected based on a soluble solid content of 20.000 ± 0.005°Brix (measured by a handheld Brix refractometer) and a pH of 4.22 ± 0.01. The selected mangoes were sanitized by immersion in sodium hypochlorite solutions (200 mg/L) for 5 min, peeled, cut into uniform pieces, and stored at −20°C until further use.

For juice preparation, the frozen mango pieces were thawed (25°C for 30 min for 1 kg of frozen mango pieces) and blended into a puree. Pectinex Ultra SP‐L was added to the puree at a concentration of 0.5% (*v*/*w*, sufficient to facilitate juice extraction by hydrolysis of pectin), and the mixture was maintained at 40°C in a water bath (JSWB‐30T; JSR, Korea) with overhead stirring set to 500 rpm (RW20 Digital; IKA, Germany) for 30 min to facilitate enzymatic breakdown of pectin. Subsequently, the temperature was raised to 80°C for 10 min to inactivate the enzyme. The resulting mixture was centrifuged to separate the juice, which was collected for further analysis.

Sucrose (37.5 g) was added to mango juice (JUICE), and the final volume was adjusted to 100 mL to obtain JUICE SU.

### 2.3. Enzymatic Synthesis of sc‐FOS and Sugar Analysis

Following pH adjustment to 5.5 with 2 M NaOH, mango juice was precisely weighed into a flask and subjected to enzymatic treatment with 3% (*v*/*w*) Pectinex Ultra SP‐L at 50°C in a shaking water bath (WNB; Memmert, Schwabach, Germany) for 0, 1, 2, 3, and 4 h. Upon completion of the enzymatic treatment, the samples were immediately subjected to thermal inactivation by immersion in boiling water for 5 min (timing started after the temperature of the mixture reached 80°C). The resulting juice was filtered through a nylon syringe filter (0.45 *μ*m) and analyzed for sugar composition using high‐performance liquid chromatography (HPLC), following the analytical conditions and instrumentation described by Kamchonemenukool et al. [2]. Conditions for the HPLC analyses were as follows: column, ZORBAX NH_2_ (5 *μ*m, 4.6 mm × 150 mm; Agilent, Santa Clara, California, United States); mobile phase, acetonitrile/water (70:30,*v*/*v*); flow rate, 1.2 mL min^−1^; temperature, 30°C; and refractive index detector 5450 (Hitachi High‐Tech Science, Tokyo, Japan).

### 2.4. OD

#### 2.4.1. Mango Preparation

The physicochemical and structural characteristics specific to each mango cultivar determine its optimal utilization. The “Nam Dok Mai” cultivar is primarily consumed fresh at full ripeness due to its notably high sugar content, which contributes to its pronounced sweetness [15]; therefore, it was selected in this study for FOS synthesis. In contrast, the “Kaew” cultivar at the mature‐green to early‐ripening stage is well suited for OD. Its densely organized pectin–cellulose cell wall matrix provides strong resistance to osmotic stress in hypertonic solutions, thereby preserving tissue integrity and crisp texture during processing [16]. Therefore, this cultivar was used for OD product development.

*M. indica* L. cv. Kaew was selected at a mature‐green stage, characterized by a pH of 3.63 ± 0.15 and a soluble solid content of 12.15 ± 1.56°Brix. Upon arrival, mangoes were sanitized by immersion in sodium hypochlorite solutions (200 mg/L) for 5 min. Following sanitization, the fruits were peeled. The peeled mangoes were then precisely cut into rectangular samples measuring 1.00 ± 0.10 cm in length, 1.00 ± 0.10 cm in width, and 0.50 ± 0.10 cm in thickness. The mango pieces were then immersed in the sc‐FOS–enriched mango syrup within glass bottles. This study investigated the impact of temperature (40°C vs. 60°C) on the sugar profile—specifically sc‐FOS, sucrose, glucose, and fructose—in mango pieces during OD. Two concentrations of sc‐FOS–enriched mango syrup, approximately 40% and 50% total sugar content, served as osmotic solutions. The ratio of mango pieces to osmotic solution was maintained at 1:5 (*w*/*v*). The OD was carried out at a controlled temperature (e.g., 40°C or 60°C) in a water bath shaker set to 120 rpm for 0, 1, 2, 3, 4, and 6 h. Samples were removed from the solution and immediately immersed in a water–ice bath for 10 s to rapidly cool the samples, minimize tissue damage, and eliminate residual osmotic solution from the surface. Subsequently, the sample surfaces were dried using absorbent paper [17].

Some mango fruits were separated for a brine‐treated process. Brine treatment of mango fruits took place in glass jars that were washed and sterilized before use in a hot air oven (150°C) for 15 min. The sanitized fruits were washed and introduced into 1500‐mL volume jars (6–7 mangoes in every jar). After that, the brine was added, just enough to cover the mangoes. The brine was prepared by boiling 1 L of limewater, prepared from 10 g of food‐grade lime in 1 L of clean water, and 100 g of food‐grade NaCl. The jars were closed and left at 27.5 ± 2.5°C for 30 days. For the brine‐treated mango, the salt removal step was conducted after the fruits were peeled and cut into pieces before the OD process by immersing the pieces in distilled water and agitating them in an orbital shaker set to 120 rpm at 25°C for 1 h.

#### 2.4.2. Analytical Measurements

Solid content of OD mango pieces was analyzed according to the AOAC official method. For the different time intervals, approximately 2–3 g of the sample was oven‐dried at 105°C until the consecutive constant weight was attained.

To determine the sugar composition of the OD mango pieces, deionized water was added to the samples at a ratio of 1:5 (*w*/*v*), based on the original weight of the mango slices prior to the OD process. The mixture was homogenized using a high‐speed blender and subsequently agitated in an orbital shaker set to 120 rpm at 25°C for 1 h. Following agitation, the mixture was centrifuged at 3000 *g* for 15 min, and the supernatant was collected. The resulting liquid was filtered through a 0.45‐*μ*m membrane filter and analyzed for sugar composition using HPLC.

At each time point, samples were collected, the surface syrup was removed as described in Section 2.4.1, and the samples were weighed. The following parameters, SG, WL, and RSM, were then calculated.

SG represented the amount of solute (from the syrup) absorbed by the fruit during OD, calculated using Equation (1). WL indicated the amount of water removed from the fruit during the process, calculated using Equation (2).

(1) SG %=mt−m0∗100m0,

(2) WL=M0−m0−Mt−mtm0,

where *m*_*t*_ is the dry mass of mango after time *t* of osmotic treatment, *m*_0_ is the dry mass of fresh material, *M*_0_ is the initial mass of fresh material before the osmotic treatment, and *M*_*t*_ is the mass of mango samples after time *t* of osmotic treatment.

RSM was simply the mass of the sample after OD, calculated using Equation (3).

(3) RSM %=Mt∗100M0.

All measurements were performed in triplicate, and results were expressed as mean ± standard deviation.

### 2.5. Scanning Electron Microscopy (SEM)

To prepare samples for drying, an ethanol series was employed for water removal. Samples were sequentially transferred through 30%, 50%, 70%, and 90% (*v*/*v*) ethanol solutions, followed by three 1‐h washes in 95% (*v*/*v*) ethanol. For complete dehydration, the ethanol‐treated samples were then freeze‐dried. Following dehydration, the dried samples were mounted and sputter‐coated with a thin layer of gold. The prepared and coated samples were subsequently observed using a LEO 1455VP SEM (Carl Zeiss, Germany).

### 2.6. Statistical Analysis

The data were analyzed using analysis of variance (ANOVA). Differences between samples were determined by the LSD test (*p* < 0.05). The Jamovi program was used for data analysis.

## 3. Results and Discussion

### 3.1. Synthesis of sc‐FOS From Mango Substrates

Fully ripe Nam Dok Mai mangoes are naturally high in sucrose and fructose, contributing to their sweet flavor. Table 1 represents the sugar composition at the initial stage of the sc‐FOS synthesis reaction (0 h). The amount of sucrose added to the JUICE resulted in a final sucrose concentration in the mango solution of approximately 500 mg/mL, which corresponds to the optimal sucrose level for sc‐FOS synthesis [3]. Using a sucrose concentration exceeding 60% for sc‐FOS synthesis results in increased viscosity, which impairs mass transfer and consequently reduces the sc‐FOS synthesis reaction rate. Furthermore, high solute concentrations lower water activity, thereby restricting the conformational flexibility of the enzyme required for optimal catalytic activity. Notably, sc‐FOS was detected in both JUICE SU and JUICE at the onset of the enzymatic sc‐FOS synthesis. This suggested that detectable amounts of sc‐FOS were produced during mango juice preparation (Section 2.2), where Pectinex Ultra SP‐L catalyzed both hydrolysis and transfructosylation. The latter occurred at a very low rate due to the limited enzyme concentration, resulting in sc‐FOS being detected in JUICE. In the case of JUICE SU during enzyme‐mediated sc‐FOS synthesis, sc‐FOS was produced rapidly because of the high sucrose content in the system. The reaction began upon enzyme–substrate contact; however, it was immediately terminated by thermal inactivation (Section 2.3), leaving only minimal sc‐FOS detected in JUICE SU at the onset of the enzymatic sc‐FOS synthesis.

**Table 1:** Sugar compositions at the initial (0 h) stage of the sc‐FOS synthesis reaction.

Sugar (mg/mL)	JUICE	JUICE SU
Glucose	35.14 ± 0.72	23.48 ± 2.07
Fructose	69.58 ± 1.71	40.91 ± 3.11
Sucrose	101.54 ± 2.17	473.56 ± 6.57
sc‐FOS	1.76 ± 0.03	46.12 ± 0.95
Total	208.02 ± 1.16	584.07 ± 3.18

**Figure 1:**
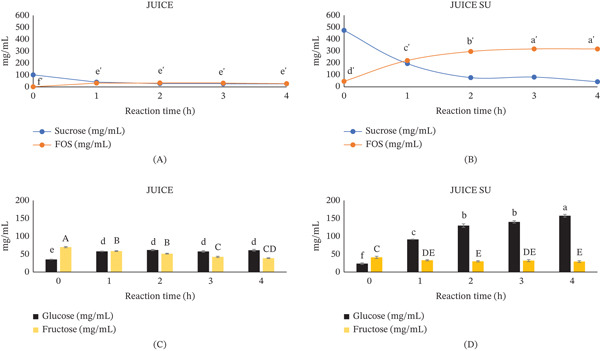
(A–D) Changes in sugar concentration during the sc‐FOS synthesis reaction. Different letters (a ^′^–f ^′^, a–e, and A–D) indicate statistically significant differences (*p* < 0.05) in sc‐FOS, glucose, and fructose concentrations among samples at different soaking times, respectively.

At the onset of sc‐FOS synthesis, the sucrose concentration in the JUICE was 102 ± 2 mg/mL. As the reaction time increased, the sc‐FOS concentration continued to rise, reaching 28 ± 1 mg/mL at 4 h of synthesis (Figure 1A). Concurrently, glucose levels rose in both substrates, indicating the enzymatic cleavage of sucrose and transfer of fructosyl units (61 ± 3 mg/mL for glucose content at 4 h of the synthesis reaction) (Figure 1C). sc‐FOS production in sucrose‐supplemented systems (JUICE SU) was markedly superior to that of the nonsupplemented (JUICE). The sucrose conversion for JUICE SU was 67.54% (g FOS·g initial sucrose^−1^), which was much higher than that of JUICE (5.98% g FOS·g initial sucrose^−1^). These observations align with the fundamental principles of enzyme kinetics, where increased substrate availability facilitates higher rates of enzymatic conversion [18]. The higher sucrose concentration of JUICE SU was a key factor supporting the greater enzymatic efficiency and subsequent sc‐FOS yield observed in this system. According to the Michaelis–Menten model, the rate of an enzyme‐catalyzed reaction increases with substrate concentration until it approaches a maximal velocity (Vmax) when the enzyme becomes saturated. In the context of this study, sucrose served as the substrate for the FTase activity that produced sc‐FOS. Therefore, higher sucrose concentrations shifted the system toward increased reaction velocity. This theoretical framework aligns with our experimental findings.

Chumjitchuen et al. [19] investigated sc‐FOS synthesis in ripe “Nam Dok Mai” mango juice using the same enzyme as in the present study. Their findings align with this study, demonstrating effective sucrose reduction and sc‐FOS production. Their reported yield of 51 mg/mL was comparable to the sc‐FOS levels produced in our nonsucrose‐supplemented systems (JUICE); however, it remained substantially lower than the yields obtained in this study through high sucrose supplementation.

Throughout the reaction, glucose levels increased with time, while fructose levels generally declined, consistent with the mechanism of transfructosylation (Figure 1C,D). During the enzymatic synthesis of sc‐FOS, the carbohydrate composition underwent a dynamic transformation characterized by a rapid depletion of sucrose alongside a simultaneous rise in sc‐FOS and glucose. As the primary substrate, sucrose exhibited a rapid depletion, serving as both the fructosyl donor and the initial acceptor for chain elongation. This consumption led to the sequential formation of sc‐FOS (sum of GF2, GF3, and GF4), which typically followed a parabolic growth curve that plateaued as the substrate was exhausted (Figure 1A,B). Simultaneously, glucose accumulated linearly as a mandatory byproduct of each transfer step. Notably, the fructose concentration remained consistently low and stable near the baseline (Figure 1C,D). This lack of accumulation served as a critical indicator of the high transfructosylating efficiency (the ratio of the FTase activity [Ut] to the hydrolase activity [Uh]) of the commercial enzymes, confirming that the fructosyl moieties were being successfully integrated into short‐chain fructans rather than being released through hydrolysis. The results of this study are consistent with those obtained when pure sucrose and sucrose‐rich raw materials were used as substrates [2, 18].

The synthesis of sc‐FOS was influenced by the initial sucrose concentration of the mango substrate. Mango syrup derived from juice with added sucrose (JUICE SU) exhibited a higher sc‐FOS content, comprising 58.07 g FOS·100 g total sugar^−1^ (the ratio of sc‐FOS:sucrose:glucose:fructose was 58:8:29:5). Conversely, substrates without supplemental sucrose resulted in substantially lower conversion rates, with sc‐FOS accounting for only 18.18 g FOS·100 g total sugar^−1^ (the ratio of sc‐FOS:sucrose:glucose:fructose was 18:17:39:25). Because the sc‐FOS–enriched mango syrup was intended for use as an osmotic solution to incorporate sc‐FOS into dehydrated mango pieces, the JUICE SU condition was selected for subsequent experiments due to its superior sc‐FOS yield.

### 3.2. Influence of Temperature and Osmotic Solution Concentration on the Sugar Profile During OD of Mango Pieces

The OD mechanism involves mass transfer phenomena driven by the difference in osmotic pressure between the food material and the surrounding osmotic solution [20, 21]. It involves three main flows: (1) WL, where water exits the fruit into the osmotic solution; (2) solid uptake, where sugars and other solutes from the solution enter the fruit; and (3) leaching, where the fruit’s own soluble compounds (like sugars, acids, and vitamins) diffuse out into the solution [20, 21]. High sugar concentration in the osmotic solution creates a stronger osmotic pressure gradient between the fruit cells and the surrounding solution. This accelerates water migration and solid uptake [21].

The FOS‐enriched mango syrup concentration was limited to 50% in relation to sc‐FOS preparation as described earlier in Section 3.1. This study focused on temperatures of 40°C and 60°C to establish a clear contrast in OD kinetics and to evaluate the distinct effects of temperature on syrup penetration. The temperature of 40°C was chosen to minimize energy consumption while still facilitating solute transfer, whereas 60°C was applied to enhance mass transfer and accelerate the dehydration process. A temperature of 60°C did not cause deterioration of textural quality in the “Kaew” cultivar due to its densely organized pectin–cellulose cell wall matrix that provides exceptional resistance to osmotic stress. By evaluating these two distinct temperature conditions, a reliable trend in OD behavior was established.

**Figure 2:**
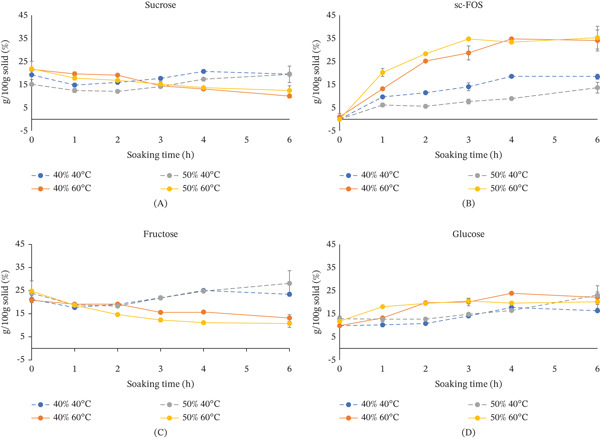
Effect of temperature on (A) sucrose, (B) sc‐FOS, (C) fructose, and (D) glucose content of mango pieces soaked in sc‐FOS–enriched mango syrup at two different concentrations, 40% and 50%. The 40% syrup contained 41 ± 2 mg/mL of fructose, 113 ± 8 mg/mL of glucose, 32.1 ± 0.9 mg/mL of sucrose, and 235 ± 15 mg/mL of sc‐FOS. The 50% syrup contained 36.1 ± 4 mg/mL of fructose, 127 ± 15 mg/mL of glucose, 73.6 ± 1.5 mg/mL of sucrose, and 293 ± 31 mg/mL of sc‐FOS.

Figure 2 shows changes in sc‐FOS uptake and uptake of other sugars by OD mango pieces soaked in sc‐FOS–enriched mango syrup consisting of 40% and 50% sugar concentrations. The OD was studied at 40°C and 60°C. The syrup concentrations used in this study were in the range commonly used for OD of mango fruits [22]. At the same treatment temperature, mango pieces exhibited similar sugar profiles after OD, regardless of syrup concentration (40% or 50%) (Figure 2A–D). Although the 50% sc‐FOS–enriched syrup contained a higher initial sc‐FOS level than the 40% syrup, after 4 h of treatment at 60°C, the equilibrium driven by a comparable osmotic pressure gradient led both syrups to achieve similar final sc‐FOS concentrations (34%–35% FOS/solid). A similar pattern was observed for lower‐molecular‐weight saccharides, including sucrose, glucose, and fructose, which showed no significant differences between treatments. These findings suggested that both syrup concentrations generated an osmotic pressure gradient sufficient to drive effective mass transfer.

Soaking temperature significantly affected sc‐FOS uptake. At 60°C, the 50% sc‐FOS–enriched mango syrup exhibited higher uptake than the 40% syrup during the first hour of soaking. This was attributed to the increased kinetic energy at 60°C, which enhanced molecular mobility and membrane permeability [20]. Consequently, the system reached equilibrium more rapidly, explaining why both treatments (40% and 50%) converged to a similar final profile after 4 h. In contrast, at 40°C, the 40% syrup resulted in greater sc‐FOS uptake than the 50% syrup. This difference was likely due to the higher viscosity of more concentrated solutions, which created external resistance at the fruit surface and reduced solute transfer. Such an effect was not observed at 60°C. Zongo et al. [12] reported that increased osmotic solution viscosity reduced sugar uptake during OD of mangoes.

Overall, both temperatures promoted sc‐FOS and glucose uptake; however, they induced distinct behaviors in sucrose and fructose. At 60°C, mango pieces exhibited more rapid sc‐FOS diffusion (Figure 2B) and a pronounced reduction in sucrose and fructose compared to 40°C (Figure 2A,C), indicating enhanced mass transfer at elevated temperatures. The sc‐FOS–enriched syrup contained a sc‐FOS:sucrose:glucose:fructose ratio of 56:8:27:10, with sc‐FOS as the predominant component. Its rapid penetration likely limited the subsequent entry of other sugars into the fruit tissue.

Conversely, at 40°C, sugar changes were more gradual, indicative of a slower dehydration process. Lower temperatures (40°C) tended to maintain or slightly increase sucrose and fructose content while still promoting sc‐FOS uptake, albeit less dramatically, probably due to the larger sc‐FOS molecules, which penetrated less into this tissue. At lower temperatures, the size of osmotic agent molecules significantly impacts solute uptake during dehydration. Bernardi et al. [11] demonstrated this by comparing inverted sugar and sucrose syrups in mango dehydration. Their study, conducted at 45°C, found that inverted sugar syrup led to a greater incorporation of solids into the mango tissue. This was because the smaller‐molecular‐weight carbohydrates in inverted sugar could penetrate the fruit’s cellular structure more easily than sucrose.

Higher temperatures significantly enhanced the penetration of solutes into fruit during OD, even for larger molecules. As temperatures increase, the kinetic energy of molecules rises, leading to more rapid movement and diffusion of solutes from the syrup into the fruit tissue [21]. This enhanced mobility facilitated the faster net movement of sugars, particularly sc‐FOS and glucose, the major components of the osmotic syrups. Previous research has demonstrated that increasing the processing temperature from 22°C to 60°C significantly enhanced the uptake of fructose, sucrose, and sc‐FOS during the OD of plums and apples [23]. Furthermore, elevated temperatures could induce structural modifications in fruit tissues, such as the softening of cell walls, the loosening of intercellular spaces, and increased cell membrane permeability resulting from altered lipid bilayer fluidity [9]. The decompartmentalization of tissues, including plasma membrane ruptures, altered the semipermeable properties of the tissue. This enhanced permeability was critical for the penetration of larger molecules like sc‐FOS, which might otherwise diffuse slowly at lower temperatures. A study by Rastogi and Raghavarao [24] on OD of pretreated potatoes reported that the breakage of cell wall structures significantly increased cell permeability, thereby accelerating mass transfer during OD.

### 3.3. OD Mango Prepared From sc‐FOS–Enriched Mango Syrup Compared to Sucrose Syrup

**Figure 3:**
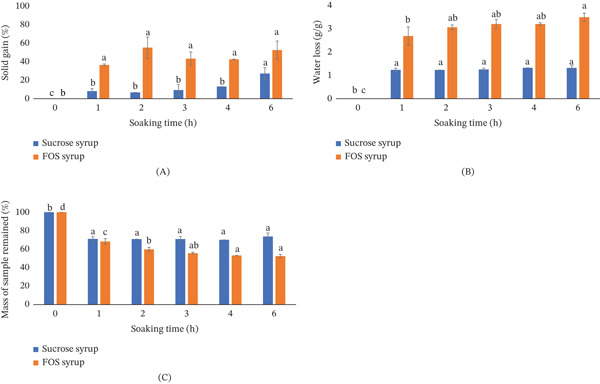
(A) Solid gain (SG), (B) water loss (WL), and (C) retained sample mass (RSM) of mango pieces soaked in sc‐FOS–enriched mango syrup or sucrose syrup at 60°C over time. sc‐FOS–enriched mango syrup contained a total sugar content of 51 ± 1 g/100 mL (27 ± 2 mg/mL of fructose, 139.6 ± 0.1 mg/mL of glucose, 47 ± 4 mg/mL of sucrose, and 295 ± 10 mg/mL of sc‐FOS). Sucrose was prepared from purified sucrose at 50 g/100 mL. Letters indicate statistically significant differences (*p* < 0.05) among soaking times for either sc‐FOS–enriched mango syrup or sucrose syrup.

The OD kinetics of mango slices in two different osmotic media are illustrated in Figure 3. Both SG and WL increased significantly during the first hour of soaking for both syrups (Figure 3A,B). However, mangoes treated with sc‐FOS–enriched mango syrup exhibited significantly higher rates of SG and WL compared to those treated with traditional sucrose syrup. This enhanced uptake of solids and removal of water indicated that the sc‐FOS–enriched mango syrup more effectively facilitated OD.

Conversely, the RSM decreased over time in both treatments (Figure 3C). However, mango pieces treated with sucrose syrup retained more mass than those in sc‐FOS–enriched mango syrup, particularly during longer soaking durations. This difference in RSM directly correlated with the lower SG and WL observed in the sucrose‐treated samples. The slower osmotic exchange in sucrose syrup, primarily involving sucrose accumulation with minimal changes in fructose and glucose content, contributed to this higher retained mass.

**Figure 4:**
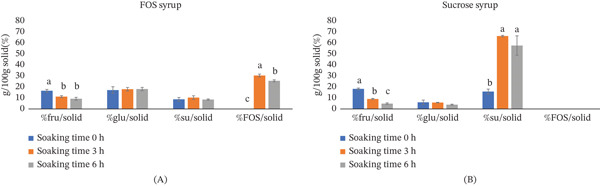
Sugar composition of mango pieces soaked in (A) sc‐FOS–enriched mango syrup or (B) sucrose syrup at 60°C over time. sc‐FOS–enriched mango syrup contained a total sugar content of 51 ± 1 g/100 mL (27 ± 2 mg/mL of fructose, 139.6 ± 0.1 mg/mL of glucose, 47 ± 4 mg/mL of sucrose, and 295 ± 10 mg/mL of sc‐FOS). Sucrose was prepared from purified sucrose at 50 g/100 mL. Letters indicate statistically significant differences (*p* < 0.05) among soaking times for either sc‐FOS–enriched mango syrup or sucrose syrup.

As reported by Nithyalakshmi and Gayathri [25], both syrup concentration and temperature were known to influence SG and WL. In this study, both FOS‐enriched mango syrup and sucrose syrup were maintained at the same concentration (50%), and OD was conducted at the same temperature (60°C). Therefore, the observed differences in SG and WL were attributed to the distinct sugar compositions of the syrups. The presence of smaller sugar molecules, such as monosaccharides like glucose and fructose, in the osmotic solute led to more rapid solute uptake and water removal. When mango juice with added sucrose to a final concentration of 50% was used as the osmotic solution, a higher reduction in RSM compared to using sucrose syrup was noticed. The RSM was 76 ± 1*%* versus 52.0 ± 0.7*%* after 1 h of soaking and 69 ± 2*%* versus 38 ± 3*%* after 3 h of soaking. This indicated that mango juice as an osmotic solute caused greater water removal. The sc‐FOS–enriched mango syrup contained a ratio of sc‐FOS:sucrose:glucose:fructose of 56:8:27:10. The monosaccharides (glucose and fructose) comprised 37% of the total sugar in the syrup, whereas the sucrose syrup consisted predominantly of disaccharides. The observed mass transfer behavior in Figure 3 is logically supported by the sugar composition analysis presented in Figure 4.

Figure 4A,B shows sugar compositions of mango pieces soaked in sc‐FOS–enriched syrup or sucrose syrup. At 3 h of soaking, monosaccharides (glucose and fructose) accounted for 29% in mangoes soaked in sc‐FOS–enriched mango syrup compared to 15% in those treated with sucrose syrup. The lower molecular weight of monosaccharides in the sc‐FOS syrup facilitated more rapid diffusion into the mango tissue while simultaneously creating a higher osmotic potential that drove water removal. This is consistent with prior studies reporting that fruit juice concentrates are more efficient osmotic agents than pure sucrose solutions due to their low‐molecular‐weight solute profiles. For example, concentrated grape juice was found to be an effective osmotic agent for yellow melon, resulting in superior WL and SG relative to sucrose [10]. The presence of glucose and fructose in most fruit concentrates created a low‐molecular‐weight osmotic medium that penetrated cellular structures more readily, thereby enhancing WL and solute uptake [11, 12].

In the sucrose‐treated samples, endogenous fructose migrated rapidly out of the tissue into the medium, while sucrose levels reached 81% of the total sugar content (Figure 4B). This high sucrose concentration often forms a solute barrier at the fruit surface, which can impede further moisture migration (lower WL). In contrast, the sc‐FOS–enriched mango syrup, which contained a higher initial concentration of fructose than the sucrose syrup, exhibited a slower reduction in fructose content. The glucose content in mango pieces was significantly higher when treated with sc‐FOS–enriched mango syrup, due to its higher initial glucose concentration. The quantitative analysis in Figure 4A confirms the successful fortification of the fruit matrix. sc‐FOS became the predominant sugar fraction in the treated mangoes, accounting for 30% (db), whereas it remained absent in the control group. In conclusion, utilizing sc‐FOS–enriched syrup derived from mango juice not only enhances processing efficiency through superior mass transfer (WL and SG) but also serves as an effective vehicle for developing functional prebiotic‐fortified fruit products.

### 3.4. Influence of Brine Treatment Before OD Using sc‐FOS–Enriched Mango Syrup

OS mango can be produced either from fresh mangoes or from brine‐treated mangoes. Brine treatment is a preservation method that allows mature green mangoes to be stored at ambient temperature for extended periods. Salt acts as a powerful natural preservative by drawing moisture out of mangoes through osmosis. It also plays a key role in suppressing undesirable microbes while allowing beneficial lactic acid bacteria to thrive, which then produce lactic acid to further preserve the mangoes [26]. Producing OS mango from brine‐treated mangoes offers an alternative approach to ensure a year‐round supply of raw material for production. Therefore, in this part of the study, mango pieces intended for OD in FOS‐enriched syrup were brine‐treated. Before using brine‐treated mangoes for OD, the excess salt must be removed. Then, the mangoes can be processed into OS mango using standard procedures.

Table 2 indicates changes in percentage SG and RSM values of mango pieces soaked for 0–3 h in sc‐FOS–enriched mango syrup. Brine treatment significantly improved the mango tissue’s ability to absorb sugar from the syrup, as evidenced by the consistently higher SG compared to the untreated ones (fresh samples). Additionally, the RSM was slightly higher for the brine‐treated samples. Brine treatment influenced the initial solid content of mango pieces after the salt removal process and prior to the osmotic treatment. Fresh mangoes had an initial solid content of 15.60 ± 0.66*%*. The brine‐treated mangoes exhibited a solid content of 21.76 ± 0.26*%* before salt removal and 12.98 ± 0.69*%* after salt removal. When fruit pieces are immersed in a salt solution (brine), water molecules migrate from the fruit’s cells (where water concentration is higher) across the semipermeable cell membranes into the more concentrated salt solution due to the osmotic pressure difference. This continuous removal of water from the fruit leads to a significant reduction in the fruit’s water content [20]. Concurrently, some water‐soluble components naturally present in the fruit’s cell sap—such as sugars, organic acids, minerals, and vitamins—can also leach out into the surrounding salt solution. This loss of internal solutes contributes to a reduction in the fruit’s total solids and enriches the brine with fruit‐derived compounds [21]. The calcium from limewater is known to interact with pectin in cell walls, forming stronger cross‐linkages that enhance firmness and help maintain structural integrity, thereby potentially reducing excessive tissue collapse during processing [22]. This likely explains why the RSM values were slightly higher for the brine‐treated samples compared to the fresh samples.

**Table 2:** Changes in percentage solid gain and retained sample mass of mango pieces soaked for 0–3 h in sc‐FOS‐enriched mango syrup.

Sample	Soaking time (h)	Solid gain (%)	Retained sample mass (%)
Fresh	0	0.00 ± 0.00^b^	100.00 ± 0.00^a^
	1	65.19 ± 0.67^a^	67.60 ± 3.48^b^
	2	67.03 ± 1.08^a^	58.49 ± 0.20^c^
	3	71.93 ± 6.02^a^	55.77 ± 1.05^c^

Brine‐treated	0	0.00 ± 0.00^d^	100.00 ± 0.00^a^
	1	88.10 ± 0.61^c^	69.37 ± 1.80^b^
	2	118.60 ± 13.06^b^	62.91 ± 1.36^b^
	3	124.65 ± 13.06^a^	62.25 ± 0.95^b^

*Note:* Data represent mean ± standard deviation. Letters indicate statistically significant differences (*p* < 0.05) among soaking times within the same sample.

**Figure 5:**
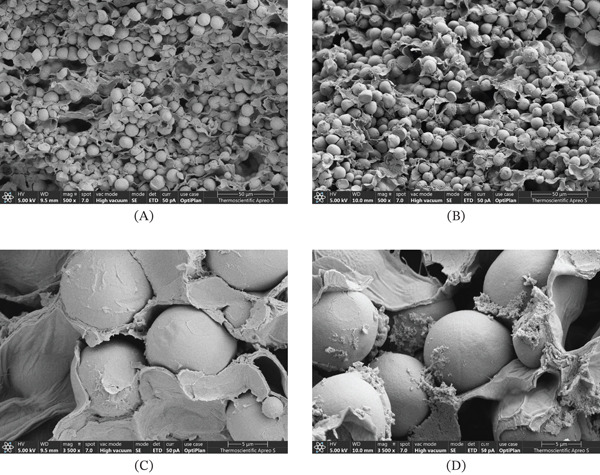
Cross‐sectional SEM images of mango cells at different magnifications. Low‐magnification views (500×) of (A) fresh and (B) brine‐treated samples. High‐magnification views (5000×) of (C) fresh and (D) brine‐treated samples.

Cross‐sectional views from SEM (Figure 5) reveal distinct microstructural differences between the brine‐treated mango samples and the fresh mango samples used for OD studies. Figure 5A displays fresh mango cells, characterized by clearly defined cell walls densely packed with starch granules. Conversely, Figure 5B, representing brine‐treated mango, exhibits cell separation. Further magnification at 5000× provides clearer evidence of cell wall alterations. Figure 5D, depicting brine‐treated mango cells, shows less dense and more porous cell walls compared to the fresh mango cells in Figure 5C. These observed modifications in cell structure, particularly the increased porosity, likely facilitated the tissue’s ability to absorb sugar from the sc‐FOS–enriched mango syrup, as evidenced by the consistently higher SG. Therefore, after the salt removal process, the brine‐treated mango tissues become more porous compared to the untreated ones, as evidenced by the SEM images (Figure 5). The increased porosity of the brine‐treated samples likely enabled greater solute uptake. As a result, the SG and mass retention of the brine‐treated samples were higher than those of the fresh samples.

## 4. Conclusions

This study successfully demonstrated a dual approach to valorize fully ripe “Nam Dok Mai” mangoes, which often contribute to significant postharvest losses, by synthesizing sc‐FOS from their sucrose content and subsequently using the sc‐FOS–enriched syrup for OD. This study established that FFase or FTase, present in commercial enzyme preparations, could effectively convert sucrose in mango juice into sc‐FOS. The addition of exogenous sucrose further enhanced sc‐FOS production. The OD experiments revealed that higher temperatures (60°C) significantly enhanced the uptake of sc‐FOS and glucose and promoted more efficient water removal and SG in mango pieces. Crucially, the sc‐FOS–enriched mango syrup was better than traditional sucrose syrup in facilitating OD, leading to greater SG and WL. The brine treatment prior to OD dramatically improved the mango tissue’s capacity for sugar absorption and retention. While this study demonstrates the feasibility of using mango‐derived sc‐FOS in OD, further assessment of product quality and stability—such as sc‐FOS stability, textural and sensory attributes, and shelf life—is recommended for future work. In conclusion, this research presented a viable and sustainable pathway for transforming unmarketable fully ripe mangoes, which commonly lead to environmental problems and economic losses for farmers due to spoilage and unmarketability, into valuable functional fruit syrup products enriched with prebiotic compounds. By converting what would otherwise be agricultural waste into sc‐FOS–enriched mango products, this study offered a solution to reduce postharvest losses and create new economic opportunities while simultaneously providing health benefits to consumers.

## Author Contributions

Jariyaporn Changin participated in data collection, data analysis, and drafting the article. Warathep Buasum participated in the analyses and drafting of the article (SEM). Sirilux Chaijamrus and Monthana Weerawatanakorn assisted in the critical revision and final approval of the version to be published. Tipawan Thongsook participated in the conception and design of the work, data analysis and interpretation, and the critical revision and final approval of the version to be published.

## Funding

This work was supported by Naresuan University (NU) and the National Science, Research and Innovation Fund (NSRF) (Grant No. R2567B092).

## Conflicts of Interest

The authors declare no conflicts of interest.

## Data Availability

The data that support the findings of this study are available from the corresponding author upon reasonable request.
